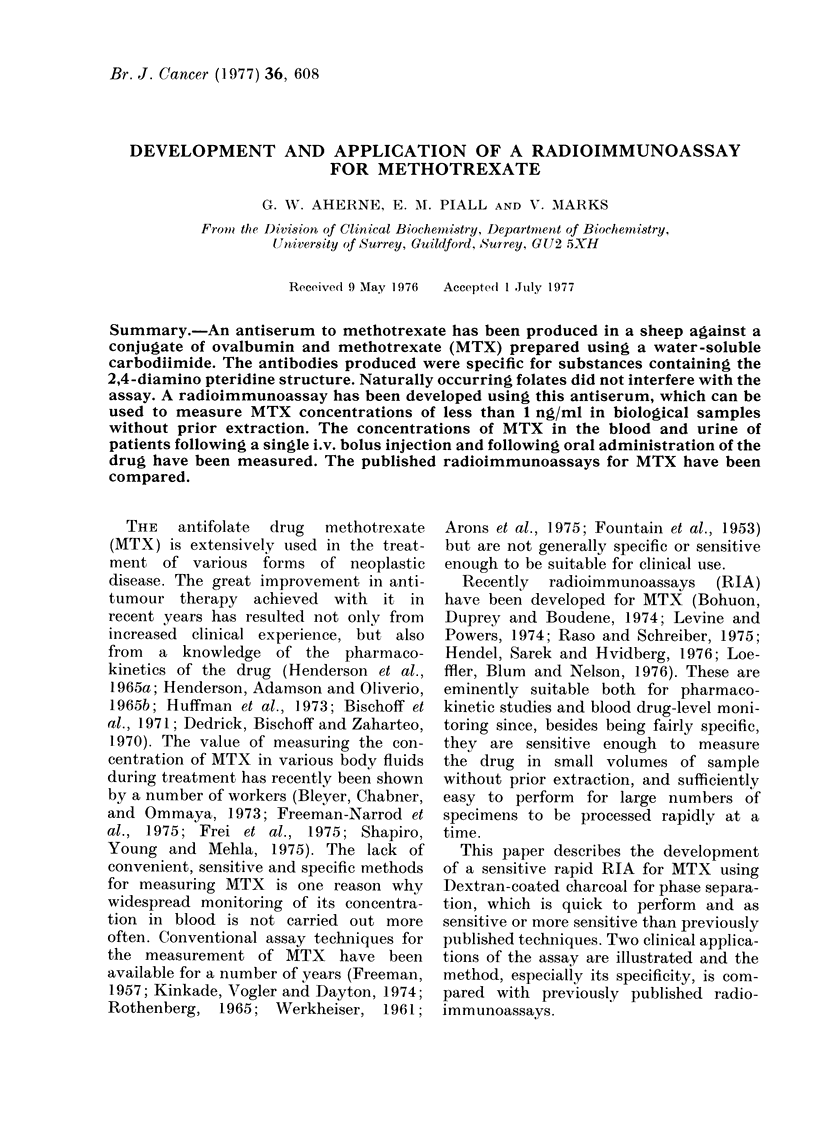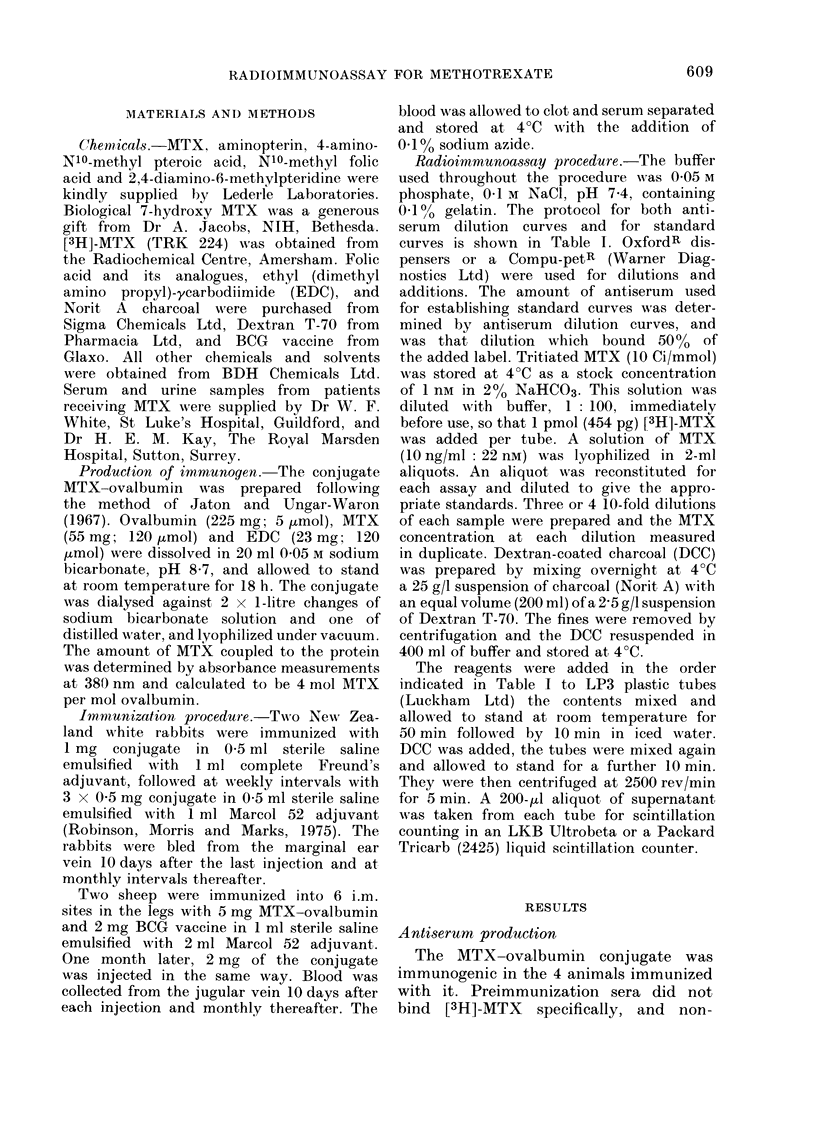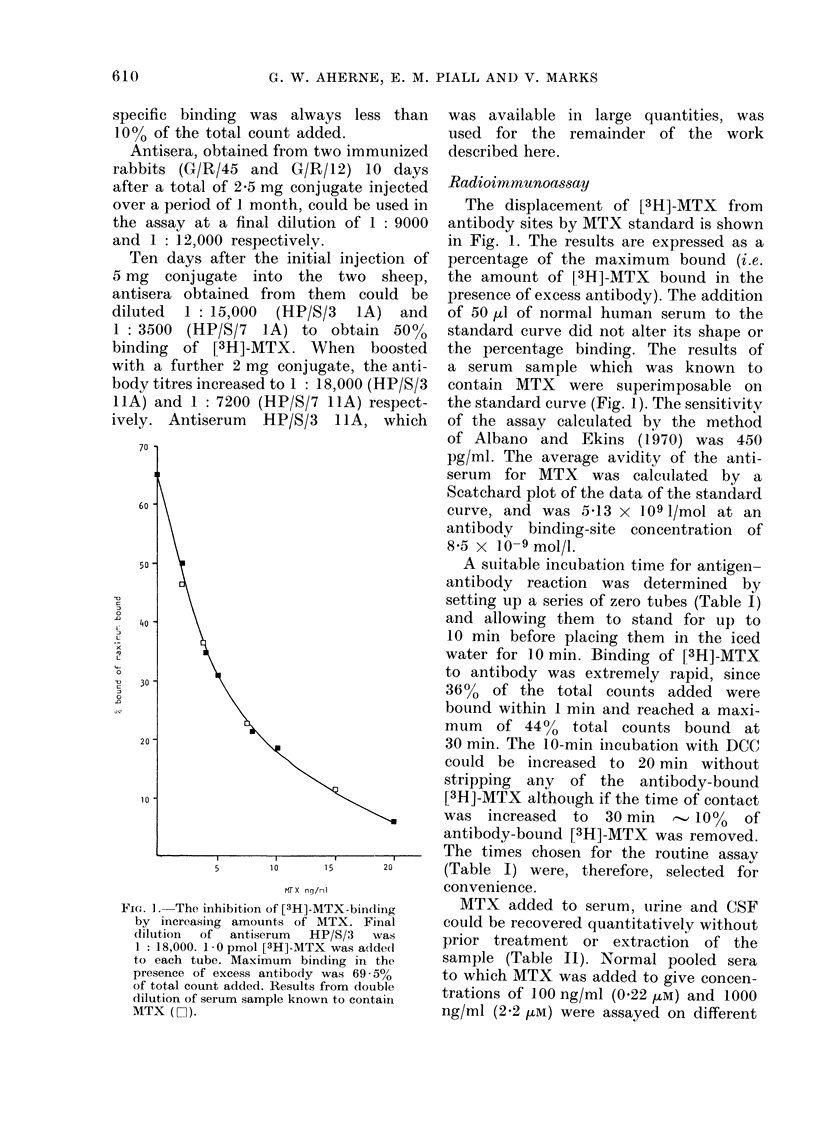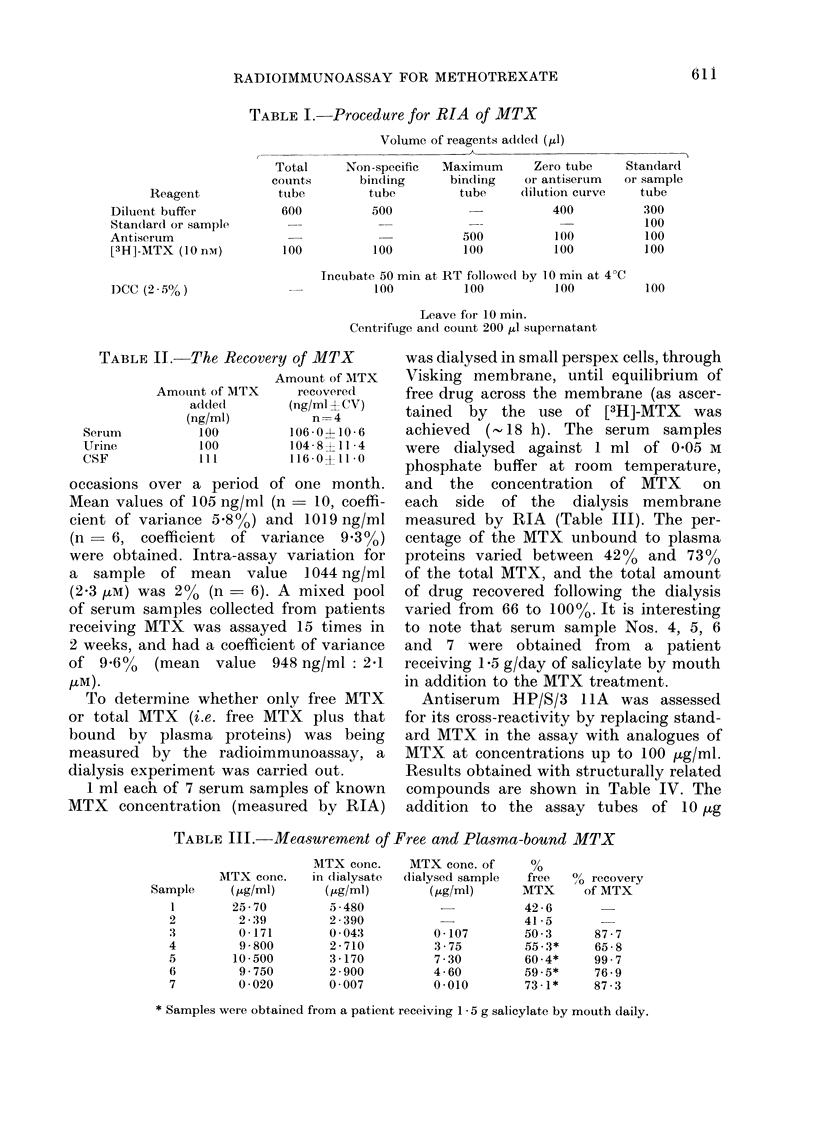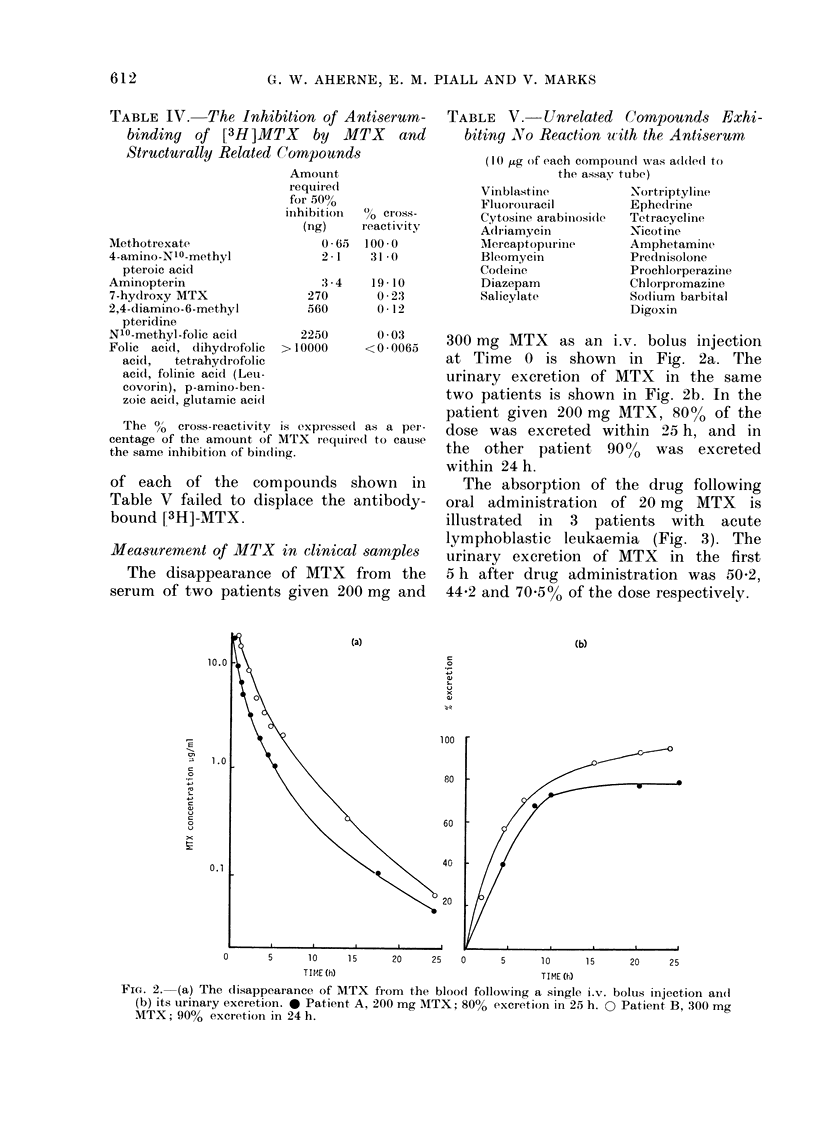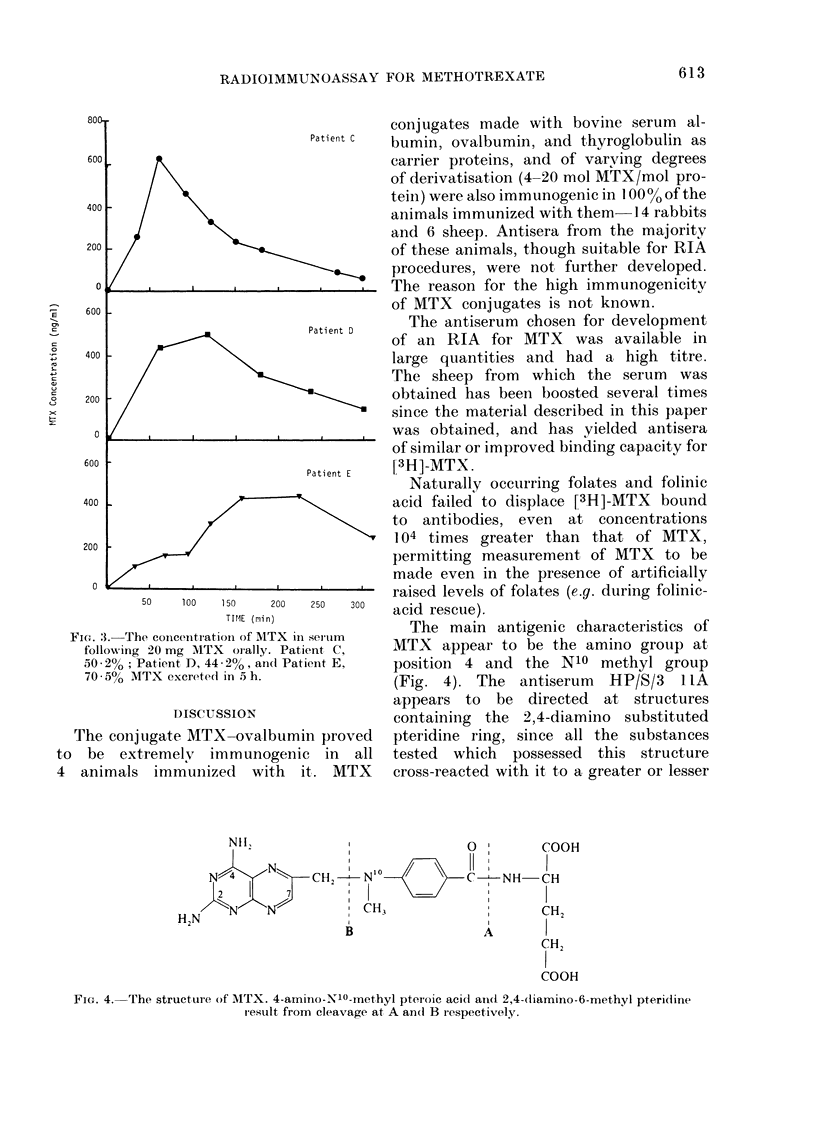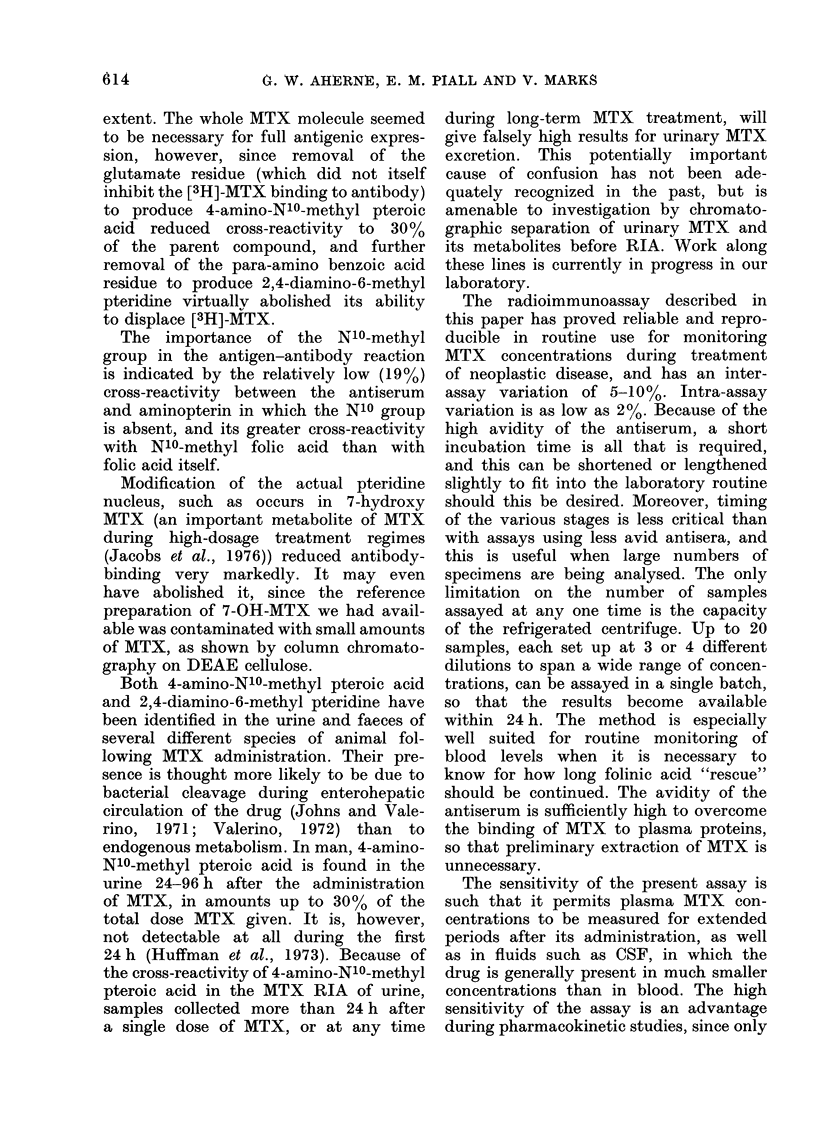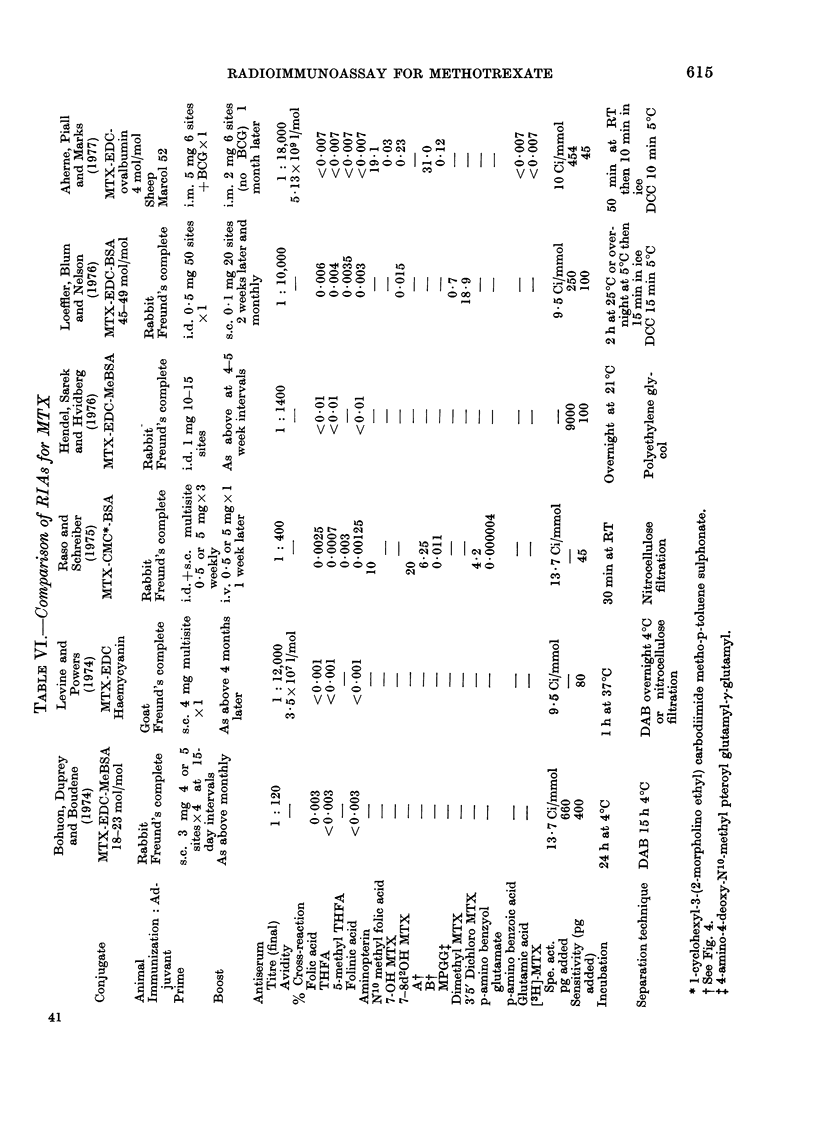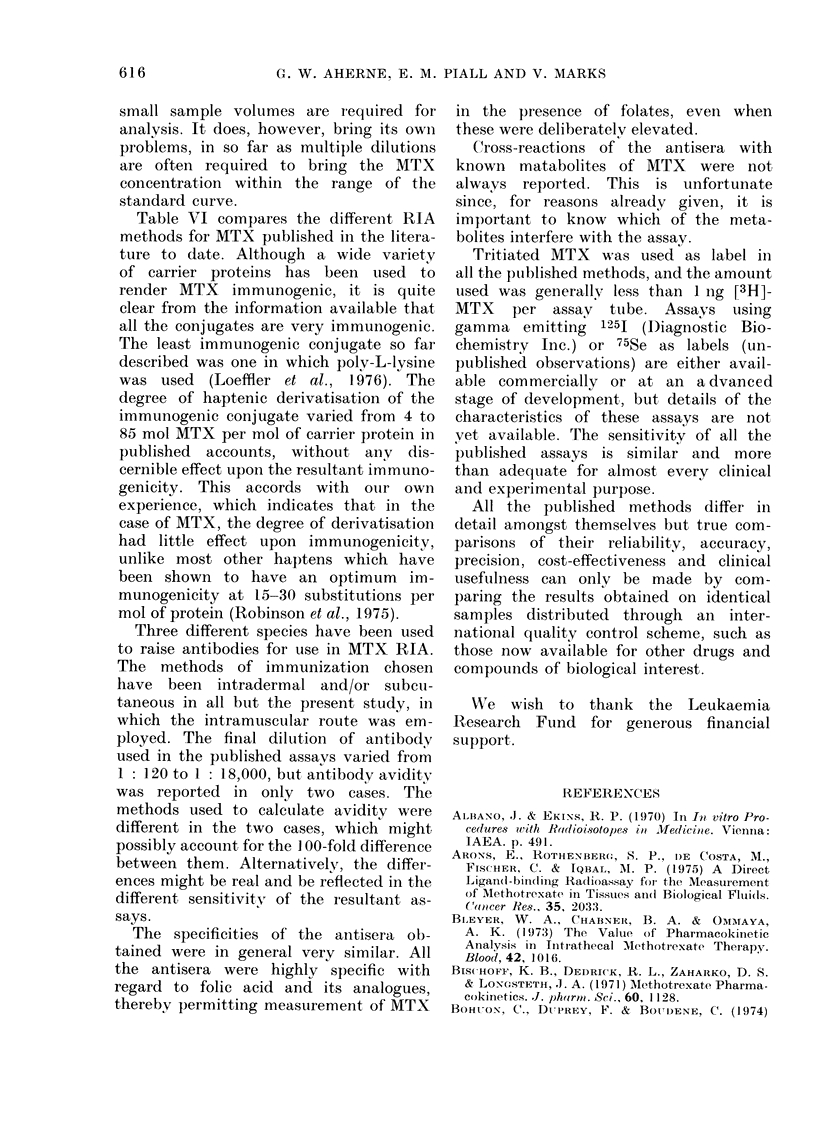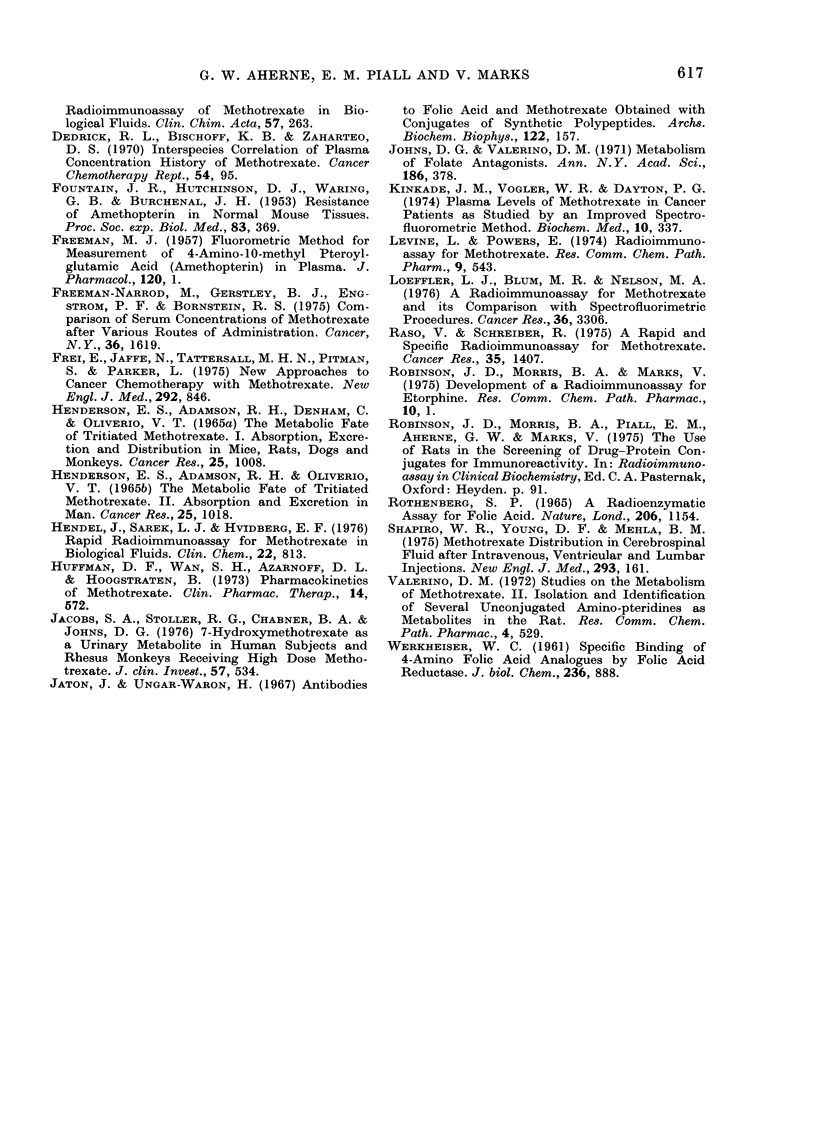# Development and application of a radioimmunoassay for methotrexate.

**DOI:** 10.1038/bjc.1977.238

**Published:** 1977-11

**Authors:** G. W. Aherne, E. M. Piall, V. Marks

## Abstract

An antiserum to methotrexate has been produced in a sheep against a conjugate of ovalbumin and methotrexate (MTX) prepared using a water-soluble carbodiimide. The antibodies produced were specific for substances containing the 2,4-diamino pteridine structure. Naturally occurring folates did not interfere with the assay. A radioimmunoassay has been developed using this antiserum, which can be used to measure MTX concentrations of less than 1 ng/ml in biological samples without prior extraction. The concentrations of MTX in the blood and urine of patients following a single i.v. bolus injection and following oral administration of the drug have been measured. The published radioimmunoassays for MTX have been compared.


Br. J. Cancer (1977) 36, 608

### DEVELOPMENT AND APPLICATION OF A RADIOIMMUNOASSAY FOR METHOTREXATE

G. W. AHERNE, E. M. PIALL AND V. MARKS

*From the Division of Clinical Biochemistry, Department of Biochemistry, University of Surrey, Guildford, Surrey, GU2 5XH*

Received 9 May 1976 Accepted 1 July 1977

**Summary.—An antiserum to methotrexate has been produced in a sheep against a conjugate of ovalbumin and methotrexate (MTX) prepared using a water-soluble carbodiimide. The antibodies produced were specific for substances containing the 2,4-diamino pteridine structure. Naturally occurring folates did not interfere with the assay. A radioimmunoassay has been developed using this antiserum, which can be used to measure MTX concentrations of less than 1 ng/ml in biological samples without prior extraction. The concentrations of MTX in the blood and urine of patients following a single i.v. bolus injection and following oral administration of the drug have been measured. The published radioimmunoassays for MTX have been compared.**

THE antifolate drug methotrexate (MTX) is extensively used in the treatment of various forms of neoplastic disease. The great improvement in anti-tumour therapy achieved with it in recent years has resulted not only from increased clinical experience, but also from a knowledge of the pharmacokinetics of the drug (Henderson *et al.*, 1965*a*; Henderson, Adamson and Oliverio, 1965*b*; Huffman *et al.*, 1973; Bischoff *et al.*, 1971; Dedrick, Bischoff and Zaharteo, 1970). The value of measuring the concentration of MTX in various body fluids during treatment has recently been shown by a number of workers (Bleyer, Chabner, and Ommaya, 1973; Freeman-Narrod *et al.*, 1975; Frei *et al.*, 1975; Shapiro, Young and Mehla, 1975). The lack of convenient, sensitive and specific methods for measuring MTX is one reason why widespread monitoring of its concentration in blood is not carried out more often. Conventional assay techniques for the measurement of MTX have been available for a number of years (Freeman, 1957; Kinkade, Vogler and Dayton, 1974; Rothenberg, 1965; Werkheiser, 1961; Arons *et al.*, 1975; Fountain *et al.*, 1953) but are not generally specific or sensitive enough to be suitable for clinical use.

Recently radioimmunoassays (RIA) have been developed for MTX (Bohuon, Duprey and Boudene, 1974; Levine and Powers, 1974; Raso and Schreiber, 1975; Hendel, Sarek and Hvidberg, 1976; Loeffler, Blum and Nelson, 1976). These are eminently suitable both for pharmacokinetic studies and blood drug-level monitoring since, besides being fairly specific, they are sensitive enough to measure the drug in small volumes of sample without prior extraction, and sufficiently easy to perform for large numbers of specimens to be processed rapidly at a time.

This paper describes the development of a sensitive rapid RIA for MTX using Dextran-coated charcoal for phase separation, which is quick to perform and as sensitive or more sensitive than previously published techniques. Two clinical applications of the assay are illustrated and the method, especially its specificity, is compared with previously published radioimmunoassays.

### MATERIALS AND METHODS

*Chemicals.*—MTX, aminopterin, 4-amino-$N^{10}$-methyl pteroic acid, $N^{10}$-methyl folic acid and 2,4-diamino-6-methylpteridine were kindly supplied by Lederle Laboratories. Biological 7-hydroxy MTX was a generous gift from Dr A. Jacobs, NIH, Bethesda. [$^3$H]-MTX (TRK 224) was obtained from the Radiochemical Centre, Amersham. Folic acid and its analogues, ethyl (dimethyl amino propyl)-$\gamma$carbodiimide (EDC), and Norit A charcoal were purchased from Sigma Chemicals Ltd, Dextran T-70 from Pharmacia Ltd, and BCG vaccine from Glaxo. All other chemicals and solvents were obtained from BDH Chemicals Ltd. Serum and urine samples from patients receiving MTX were supplied by Dr W. F. White, St Luke's Hospital, Guildford, and Dr H. E. M. Kay, The Royal Marsden Hospital, Sutton, Surrey.

*Production of immunogen.*—The conjugate MTX–ovalbumin was prepared following the method of Jaton and Ungar-Waron (1967). Ovalbumin (225 mg; 5 $\mu$mol), MTX (55 mg; 120 $\mu$mol) and EDC (23 mg; 120 $\mu$mol) were dissolved in 20 ml 0·05 M sodium bicarbonate, pH 8·7, and allowed to stand at room temperature for 18 h. The conjugate was dialysed against 2 × 1-litre changes of sodium bicarbonate solution and one of distilled water, and lyophilized under vacuum. The amount of MTX coupled to the protein was determined by absorbance measurements at 380 nm and calculated to be 4 mol MTX per mol ovalbumin.

*Immunization procedure.*—Two New Zealand white rabbits were immunized with 1 mg conjugate in 0·5 ml sterile saline emulsified with 1 ml complete Freund's adjuvant, followed at weekly intervals with 3 × 0·5 mg conjugate in 0·5 ml sterile saline emulsified with 1 ml Marcol 52 adjuvant (Robinson, Morris and Marks, 1975). The rabbits were bled from the marginal ear vein 10 days after the last injection and at monthly intervals thereafter.

Two sheep were immunized into 6 i.m. sites in the legs with 5 mg MTX–ovalbumin and 2 mg BCG vaccine in 1 ml sterile saline emulsified with 2 ml Marcol 52 adjuvant. One month later, 2 mg of the conjugate was injected in the same way. Blood was collected from the jugular vein 10 days after each injection and monthly thereafter. The blood was allowed to clot and serum separated and stored at 4°C with the addition of 0·1% sodium azide.

*Radioimmunoassay procedure.*—The buffer used throughout the procedure was 0·05 M phosphate, 0·1 M NaCl, pH 7·4, containing 0·1% gelatin. The protocol for both antiserum dilution curves and for standard curves is shown in Table I. Oxford$^R$ dispensers or a Compu-pet$^R$ (Warner Diagnostics Ltd) were used for dilutions and additions. The amount of antiserum used for establishing standard curves was determined by antiserum dilution curves, and was that dilution which bound 50% of the added label. Tritiated MTX (10 Ci/mmol) was stored at 4°C as a stock concentration of 1 nM in 2% $NaHCO_3$. This solution was diluted with buffer, 1 : 100, immediately before use, so that 1 pmol (454 pg) [$^3$H]-MTX was added per tube. A solution of MTX (10 ng/ml : 22 nM) was lyophilized in 2-ml aliquots. An aliquot was reconstituted for each assay and diluted to give the appropriate standards. Three or 4 10-fold dilutions of each sample were prepared and the MTX concentration at each dilution measured in duplicate. Dextran-coated charcoal (DCC) was prepared by mixing overnight at 4°C a 25 g/l suspension of charcoal (Norit A) with an equal volume (200 ml) of a 2·5 g/l suspension of Dextran T-70. The fines were removed by centrifugation and the DCC resuspended in 400 ml of buffer and stored at 4°C.

The reagents were added in the order indicated in Table I to LP3 plastic tubes (Luckham Ltd) the contents mixed and allowed to stand at room temperature for 50 min followed by 10 min in iced water. DCC was added, the tubes were mixed again and allowed to stand for a further 10 min. They were then centrifuged at 2500 rev/min for 5 min. A 200-$\mu$l aliquot of supernatant was taken from each tube for scintillation counting in an LKB Ultrobeta or a Packard Tricarb (2425) liquid scintillation counter.

### RESULTS

### *Antiserum production*

The MTX–ovalbumin conjugate was immunogenic in the 4 animals immunized with it. Preimmunization sera did not bind [$^3$H]-MTX specifically, and nonspecific binding was always less than 10% of the total count added.

Antisera, obtained from two immunized rabbits (G/R/45 and G/R/12) 10 days after a total of 2·5 mg conjugate injected over a period of 1 month, could be used in the assay at a final dilution of 1 : 9000 and 1 : 12,000 respectively.

Ten days after the initial injection of 5 mg conjugate into the two sheep, antisera obtained from them could be diluted 1 : 15,000 (HP/S/3 1A) and 1 : 3500 (HP/S/7 1A) to obtain 50% binding of [$^3$H]-MTX. When boosted with a further 2 mg conjugate, the antibody titres increased to 1 : 18,000 (HP/S/3 11A) and 1 : 7200 (HP/S/7 11A) respectively. Antiserum HP/S/3 11A, which

FIG. 1.—The inhibition of [$^3$H]-MTX-binding by increasing amounts of MTX. Final dilution of antiserum HP/S/3 was 1 : 18,000. 1·0 pmol [$^3$H]-MTX was added to each tube. Maximum binding in the presence of excess antibody was 69·5% of total count added. Results from double dilution of serum sample known to contain MTX (□).

was available in large quantities, was used for the remainder of the work described here.

*Radioimmunoassay*

The displacement of [$^3$H]-MTX from antibody sites by MTX standard is shown in Fig. 1. The results are expressed as a percentage of the maximum bound (*i.e.* the amount of [$^3$H]-MTX bound in the presence of excess antibody). The addition of 50 $\mu$l of normal human serum to the standard curve did not alter its shape or the percentage binding. The results of a serum sample which was known to contain MTX were superimposable on the standard curve (Fig. 1). The sensitivity of the assay calculated by the method of Albano and Ekins (1970) was 450 pg/ml. The average avidity of the antiserum for MTX was calculated by a Scatchard plot of the data of the standard curve, and was $5{\cdot}13 \times 10^9$ l/mol at an antibody binding-site concentration of $8{\cdot}5 \times 10^{-9}$ mol/l.

A suitable incubation time for antigen–antibody reaction was determined by setting up a series of zero tubes (Table I) and allowing them to stand for up to 10 min before placing them in the iced water for 10 min. Binding of [$^3$H]-MTX to antibody was extremely rapid, since 36% of the total counts added were bound within 1 min and reached a maximum of 44% total counts bound at 30 min. The 10-min incubation with DCC could be increased to 20 min without stripping any of the antibody-bound [$^3$H]-MTX although if the time of contact was increased to 30 min $\sim$10% of antibody-bound [$^3$H]-MTX was removed. The times chosen for the routine assay (Table I) were, therefore, selected for convenience.

MTX added to serum, urine and CSF could be recovered quantitatively without prior treatment or extraction of the sample (Table II). Normal pooled sera to which MTX was added to give concentrations of 100 ng/ml (0·22 $\mu$M) and 1000 ng/ml (2·2 $\mu$M) were assayed on different

TABLE I.—*Procedure for RIA of MTX*

| Reagent | Total counts tube | Non-specific binding tube | Maximum binding tube | Zero tube or antiserum dilution curve | Standard or sample tube |
|---|---|---|---|---|---|
| | Volume of reagents added (μl) | | | | |
| Diluent buffer | 600 | 500 | — | 400 | 300 |
| Standard or sample | — | — | — | — | 100 |
| Antiserum | — | — | 500 | 100 | 100 |
| [$^3$H]-MTX (10 nM) | 100 | 100 | 100 | 100 | 100 |
| | Incubate 50 min at RT followed by 10 min at 4°C | | | | |
| DCC (2·5%) | — | 100 | 100 | 100 | 100 |
| | Leave for 10 min. Centrifuge and count 200 μl supernatant | | | | |

TABLE II.—*The Recovery of MTX*

| | Amount of MTX added (ng/ml) | Amount of MTX recovered (ng/ml ± CV) n = 4 |
|---|---|---|
| Serum | 100 | 106·0 ± 10·6 |
| Urine | 100 | 104·8 ± 11·4 |
| CSF | 111 | 116·0 ± 11·0 |

occasions over a period of one month. Mean values of 105 ng/ml (n = 10, coefficient of variance 5·8%) and 1019 ng/ml (n = 6, coefficient of variance 9·3%) were obtained. Intra-assay variation for a sample of mean value 1044 ng/ml (2·3 μM) was 2% (n = 6). A mixed pool of serum samples collected from patients receiving MTX was assayed 15 times in 2 weeks, and had a coefficient of variance of 9·6% (mean value 948 ng/ml : 2·1 μM).

To determine whether only free MTX or total MTX (*i.e.* free MTX plus that bound by plasma proteins) was being measured by the radioimmunoassay, a dialysis experiment was carried out.

1 ml each of 7 serum samples of known MTX concentration (measured by RIA) was dialysed in small perspex cells, through Visking membrane, until equilibrium of free drug across the membrane (as ascertained by the use of [$^3$H]-MTX was achieved (~18 h). The serum samples were dialysed against 1 ml of 0·05 M phosphate buffer at room temperature, and the concentration of MTX on each side of the dialysis membrane measured by RIA (Table III). The percentage of the MTX unbound to plasma proteins varied between 42% and 73% of the total MTX, and the total amount of drug recovered following the dialysis varied from 66 to 100%. It is interesting to note that serum sample Nos. 4, 5, 6 and 7 were obtained from a patient receiving 1·5 g/day of salicylate by mouth in addition to the MTX treatment.

Antiserum HP/S/3 11A was assessed for its cross-reactivity by replacing standard MTX in the assay with analogues of MTX at concentrations up to 100 μg/ml. Results obtained with structurally related compounds are shown in Table IV. The addition to the assay tubes of 10 μg

TABLE III.—*Measurement of Free and Plasma-bound MTX*

| Sample | MTX conc. (μg/ml) | MTX conc. in dialysate (μg/ml) | MTX conc. of dialysed sample (μg/ml) | % free MTX | % recovery of MTX |
|---|---|---|---|---|---|
| 1 | 25·70 | 5·480 | — | 42·6 | — |
| 2 | 2·39 | 2·390 | — | 41·5 | — |
| 3 | 0·171 | 0·043 | 0·107 | 50·3 | 87·7 |
| 4 | 9·800 | 2·710 | 3·75 | 55·3* | 65·8 |
| 5 | 10·500 | 3·170 | 7·30 | 60·4* | 99·7 |
| 6 | 9·750 | 2·900 | 4·60 | 59·5* | 76·9 |
| 7 | 0·020 | 0·007 | 0·010 | 73·1* | 87·3 |

* Samples were obtained from a patient receiving 1·5 g salicylate by mouth daily.

TABLE IV.—*The Inhibition of Antiserum-binding of [$^{3}H$]MTX by MTX and Structurally Related Compounds*

| | Amount required for 50% inhibition (ng) | % cross-reactivity |
|---|---|---|
| Methotrexate | 0·65 | 100·0 |
| 4-amino-$N^{10}$-methyl pteroic acid | 2·1 | 31·0 |
| Aminopterin | 3·4 | 19·10 |
| 7-hydroxy MTX | 270 | 0·23 |
| 2,4-diamino-6-methyl pteridine | 560 | 0·12 |
| $N^{10}$-methyl-folic acid | 2250 | 0·03 |
| Folic acid, dihydrofolic acid, tetrahydrofolic acid, folinic acid (Leucovorin), p-amino-benzoic acid, glutamic acid | >10000 | <0·0065 |

The % cross-reactivity is expressed as a percentage of the amount of MTX required to cause the same inhibition of binding.

of each of the compounds shown in Table V failed to displace the antibody-bound [$^{3}$H]-MTX.

*Measurement of MTX in clinical samples*

The disappearance of MTX from the serum of two patients given 200 mg and 300 mg MTX as an i.v. bolus injection at Time 0 is shown in Fig. 2a. The urinary excretion of MTX in the same two patients is shown in Fig. 2b. In the patient given 200 mg MTX, 80% of the dose was excreted within 25 h, and in the other patient 90% was excreted within 24 h.

TABLE V.—*Unrelated Compounds Exhibiting No Reaction with the Antiserum*

(10 μg of each compound was added to the assay tube)

| | |
|---|---|
| Vinblastine | Nortriptyline |
| Fluorouracil | Ephedrine |
| Cytosine arabinoside | Tetracycline |
| Adriamycin | Nicotine |
| Mercaptopurine | Amphetamine |
| Bleomycin | Prednisolone |
| Codeine | Prochlorperazine |
| Diazepam | Chlorpromazine |
| Salicylate | Sodium barbital |
| | Digoxin |

The absorption of the drug following oral administration of 20 mg MTX is illustrated in 3 patients with acute lymphoblastic leukaemia (Fig. 3). The urinary excretion of MTX in the first 5 h after drug administration was 50·2, 44·2 and 70·5% of the dose respectively.

FIG. 2.—(a) The disappearance of MTX from the blood following a single i.v. bolus injection and (b) its urinary excretion. ● Patient A, 200 mg MTX; 80% excretion in 25 h. ○ Patient B, 300 mg MTX; 90% excretion in 24 h.

FIG. 3.—The concentration of MTX in serum following 20 mg MTX orally. Patient C, 50·2% ; Patient D, 44·2%, and Patient E, 70·5% MTX excreted in 5 h.

### DISCUSSION

The conjugate MTX–ovalbumin proved to be extremely immunogenic in all 4 animals immunized with it. MTX conjugates made with bovine serum albumin, ovalbumin, and thyroglobulin as carrier proteins, and of varying degrees of derivatisation (4–20 mol MTX/mol protein) were also immunogenic in 100% of the animals immunized with them—14 rabbits and 6 sheep. Antisera from the majority of these animals, though suitable for RIA procedures, were not further developed. The reason for the high immunogenicity of MTX conjugates is not known.

The antiserum chosen for development of an RIA for MTX was available in large quantities and had a high titre. The sheep from which the serum was obtained has been boosted several times since the material described in this paper was obtained, and has yielded antisera of similar or improved binding capacity for [$^3$H]-MTX.

Naturally occurring folates and folinic acid failed to displace [$^3$H]-MTX bound to antibodies, even at concentrations $10^4$ times greater than that of MTX, permitting measurement of MTX to be made even in the presence of artificially raised levels of folates (*e.g.* during folinic-acid rescue).

The main antigenic characteristics of MTX appear to be the amino group at position 4 and the $N^{10}$ methyl group (Fig. 4). The antiserum HP/S/3 11A appears to be directed at structures containing the 2,4-diamino substituted pteridine ring, since all the substances tested which possessed this structure cross-reacted with it to a greater or lesser

FIG. 4.—The structure of MTX. 4-amino-$N^{10}$-methyl pteroic acid and 2,4-diamino-6-methyl pteridine result from cleavage at A and B respectively.

extent. The whole MTX molecule seemed to be necessary for full antigenic expression, however, since removal of the glutamate residue (which did not itself inhibit the [$^3$H]-MTX binding to antibody) to produce 4-amino-$N^{10}$-methyl pteroic acid reduced cross-reactivity to 30% of the parent compound, and further removal of the para-amino benzoic acid residue to produce 2,4-diamino-6-methyl pteridine virtually abolished its ability to displace [$^3$H]-MTX.

The importance of the $N^{10}$-methyl group in the antigen–antibody reaction is indicated by the relatively low (19%) cross-reactivity between the antiserum and aminopterin in which the $N^{10}$ group is absent, and its greater cross-reactivity with $N^{10}$-methyl folic acid than with folic acid itself.

Modification of the actual pteridine nucleus, such as occurs in 7-hydroxy MTX (an important metabolite of MTX during high-dosage treatment regimes (Jacobs *et al.*, 1976)) reduced antibody-binding very markedly. It may even have abolished it, since the reference preparation of 7-OH-MTX we had available was contaminated with small amounts of MTX, as shown by column chromatography on DEAE cellulose.

Both 4-amino-$N^{10}$-methyl pteroic acid and 2,4-diamino-6-methyl pteridine have been identified in the urine and faeces of several different species of animal following MTX administration. Their presence is thought more likely to be due to bacterial cleavage during enterohepatic circulation of the drug (Johns and Valerino, 1971; Valerino, 1972) than to endogenous metabolism. In man, 4-amino-$N^{10}$-methyl pteroic acid is found in the urine 24–96 h after the administration of MTX, in amounts up to 30% of the total dose MTX given. It is, however, not detectable at all during the first 24 h (Huffman *et al.*, 1973). Because of the cross-reactivity of 4-amino-$N^{10}$-methyl pteroic acid in the MTX RIA of urine, samples collected more than 24 h after a single dose of MTX, or at any time during long-term MTX treatment, will give falsely high results for urinary MTX excretion. This potentially important cause of confusion has not been adequately recognized in the past, but is amenable to investigation by chromatographic separation of urinary MTX and its metabolites before RIA. Work along these lines is currently in progress in our laboratory.

The radioimmunoassay described in this paper has proved reliable and reproducible in routine use for monitoring MTX concentrations during treatment of neoplastic disease, and has an inter-assay variation of 5–10%. Intra-assay variation is as low as 2%. Because of the high avidity of the antiserum, a short incubation time is all that is required, and this can be shortened or lengthened slightly to fit into the laboratory routine should this be desired. Moreover, timing of the various stages is less critical than with assays using less avid antisera, and this is useful when large numbers of specimens are being analysed. The only limitation on the number of samples assayed at any one time is the capacity of the refrigerated centrifuge. Up to 20 samples, each set up at 3 or 4 different dilutions to span a wide range of concentrations, can be assayed in a single batch, so that the results become available within 24 h. The method is especially well suited for routine monitoring of blood levels when it is necessary to know for how long folinic acid "rescue" should be continued. The avidity of the antiserum is sufficiently high to overcome the binding of MTX to plasma proteins, so that preliminary extraction of MTX is unnecessary.

The sensitivity of the present assay is such that it permits plasma MTX concentrations to be measured for extended periods after its administration, as well as in fluids such as CSF, in which the drug is generally present in much smaller concentrations than in blood. The high sensitivity of the assay is an advantage during pharmacokinetic studies, since only

TABLE VI.—*Comparison of RIAs for MTX*

| | Bohuon, Duprey and Boudene (1974) | Levine and Powers (1974) | Raso and Schreiber (1975) | Hendel, Sarek and Hvidberg (1976) | Loeffler, Blum and Nelson (1976) | Aherne, Piall and Marks (1977) |
|---|---|---|---|---|---|---|
| Conjugate | MTX-EDC-MeBSA 18–23 mol/mol | MTX-EDC Haemycyanin | MTX-CMC*-BSA | MTX-EDC-MeBSA | MTX-EDC-BSA 45–49 mol/mol | MTX-EDC-ovalbumin 4 mol/mol |
| Animal | Rabbit | Goat | Rabbit | Rabbit | Rabbit | Sheep |
| Immunization : Adjuvant | Freund's complete | Freund's complete | Freund's complete | Freund's complete | Freund's complete | Marcol 52 |
| Prime | s.c. 3 mg 4 or 5 sites × 4 at 15-day intervals | s.c. 4 mg multisite × 1 | i.d.+s.c. multisite 0·5 or 5 mg × 3 weekly | i.d. 1 mg 10–15 sites | i.d. 0·5 mg 50 sites × 1 | i.m. 5 mg 6 sites + BCG × 1 |
| Boost | As above monthly | As above 4 months later | i.v. 0·5 or 5 mg × 1 1 week later | As above at 4–5 week intervals | s.c. 0·1 mg 20 sites 2 weeks later and monthly | i.m. 2 mg 6 sites (no BCG) 1 month later |
| Antiserum | | | | | | |
| Titre (final) | 1 : 120 | 1 : 12,000 | 1 : 400 | 1 : 1400 | 1 : 10,000 | 1 : 18,000 |
| Avidity | — | 3·5 × $10^7$ l/mol | — | — | — | 5·13 × $10^9$ l/mol |
| % Cross-reaction | | | | | | |
| Folic acid | 0·003 | <0·001 | 0·0025 | <0·01 | 0·006 | <0·007 |
| THFA | <0·003 | <0·001 | 0·0007 | <0·01 | 0·004 | <0·007 |
| 5-methyl THFA | — | — | 0·003 | — | 0·0035 | <0·007 |
| Folinic acid | <0·003 | <0·001 | 0·00125 | <0·01 | 0·003 | <0·007 |
| Aminopterin | — | — | 10 | — | — | 19·1 |
| $N^{10}$ methyl folic acid | — | — | — | — | — | 0·03 |
| 7-OH MTX | — | — | — | — | 0·015 | 0·23 |
| 7–8d²OH MTX | — | — | 20 | — | — | — |
| A† | — | — | 6·25 | — | — | 31·0 |
| B† | — | — | 0·011 | — | — | 0·12 |
| MPGG‡ | — | — | — | — | 0·7 | — |
| Dimethyl MTX | — | — | — | — | 18·9 | — |
| 3′5′ Dichloro MTX | — | — | 4·2 | — | — | — |
| p-amino benzyol glutamate | — | — | 0·000004 | — | — | — |
| p-amino benzoic acid | — | — | — | — | — | <0·007 |
| Glutamic acid | — | — | — | — | — | <0·007 |
| [³H]-MTX | | | | | | |
| Spe. act. | 13·7 Ci/mmol | 9·5 Ci/mmol | 13·7 Ci/mmol | — | 9·5 Ci/mmol | 10 Ci/mmol |
| pg added | 660 | — | — | 9000 | 250 | 454 |
| Sensitivity (pg added) | 400 | 80 | 45 | 100 | 100 | 45 |
| Incubation | 24 h at 4°C | 1 h at 37°C | 30 min at RT | Overnight at 21°C | 2 h at 25°C or overnight at 5°C then 15 min in ice | 50 min at RT then 10 min in ice |
| Separation technique | DAB 15 h 4°C | DAB overnight 4°C or nitrocellulose filtration | Nitrocellulose filtration | Polyethylene glycol | DCC 15 min 5°C | DCC 10 min 5°C |

* 1-cyclohexyl-3-(2-morpholino ethyl) carbodiimide metho-p-toluene sulphonate.

† See Fig. 4.

‡ 4-amino-4-deoxy-$N^{10}$-methyl pteroyl glutamyl-γ-glutamyl.

small sample volumes are required for analysis. It does, however, bring its own problems, in so far as multiple dilutions are often required to bring the MTX concentration within the range of the standard curve.

Table VI compares the different RIA methods for MTX published in the literature to date. Although a wide variety of carrier proteins has been used to render MTX immunogenic, it is quite clear from the information available that all the conjugates are very immunogenic. The least immunogenic conjugate so far described was one in which poly-L-lysine was used (Loeffler *et al.*, 1976). The degree of haptenic derivatisation of the immunogenic conjugate varied from 4 to 85 mol MTX per mol of carrier protein in published accounts, without any discernible effect upon the resultant immunogenicity. This accords with our own experience, which indicates that in the case of MTX, the degree of derivatisation had little effect upon immunogenicity, unlike most other haptens which have been shown to have an optimum immunogenicity at 15–30 substitutions per mol of protein (Robinson *et al.*, 1975).

Three different species have been used to raise antibodies for use in MTX RIA. The methods of immunization chosen have been intradermal and/or subcutaneous in all but the present study, in which the intramuscular route was employed. The final dilution of antibody used in the published assays varied from 1 : 120 to 1 : 18,000, but antibody avidity was reported in only two cases. The methods used to calculate avidity were different in the two cases, which might possibly account for the 100-fold difference between them. Alternatively, the differences might be real and be reflected in the different sensitivity of the resultant assays.

The specificities of the antisera obtained were in general very similar. All the antisera were highly specific with regard to folic acid and its analogues, thereby permitting measurement of MTX in the presence of folates, even when these were deliberately elevated.

Cross-reactions of the antisera with known matabolites of MTX were not always reported. This is unfortunate since, for reasons already given, it is important to know which of the metabolites interfere with the assay.

Tritiated MTX was used as label in all the published methods, and the amount used was generally less than 1 ng [$^3$H]-MTX per assay tube. Assays using gamma emitting $^{125}$I (Diagnostic Biochemistry Inc.) or $^{75}$Se as labels (unpublished observations) are either available commercially or at an a dvanced stage of development, but details of the characteristics of these assays are not yet available. The sensitivity of all the published assays is similar and more than adequate for almost every clinical and experimental purpose.

All the published methods differ in detail amongst themselves but true comparisons of their reliability, accuracy, precision, cost-effectiveness and clinical usefulness can only be made by comparing the results obtained on identical samples distributed through an international quality control scheme, such as those now available for other drugs and compounds of biological interest.

We wish to thank the Leukaemia Research Fund for generous financial support.

### REFERENCES

ALBANO, J. & EKINS, R. P. (1970) In *In vitro Procedures with Radioisotopes in Medicine.* Vienna: IAEA. p. 491.

ARONS, E., ROTHENBERG, S. P., DE COSTA, M., FISCHER, C. & IQBAL, M. P. (1975) A Direct Ligand-binding Radioassay for the Measurement of Methotrexate in Tissues and Biological Fluids. *Cancer Res.*, **35**, 2033.

BLEYER, W. A., CHABNER, B. A. & OMMAYA, A. K. (1973) The Value of Pharmacokinetic Analysis in Intrathecal Methotrexate Therapy. *Blood*, **42**, 1016.

BISCHOFF, K. B., DEDRICK, R. L., ZAHARKO, D. S. & LONGSTETH, J. A. (1971) Methotrexate Pharmacokinetics. *J. pharm. Sci.*, **60**, 1128.

BOHUON, C., DUPREY, F. & BOUDENE, C. (1974)

Radioimmunoassay of Methotrexate in Biological Fluids. *Clin. Chim. Acta*, **57**, 263.

DEDRICK, R. L., BISCHOFF, K. B. & ZAHARTEO, D. S. (1970) Interspecies Correlation of Plasma Concentration History of Methotrexate. *Cancer Chemotherapy Rept.*, **54**, 95.

FOUNTAIN, J. R., HUTCHINSON, D. J., WARING, G. B. & BURCHENAL, J. H. (1953) Resistance of Amethopterin in Normal Mouse Tissues. *Proc. Soc. exp. Biol. Med.*, **83**, 369.

FREEMAN, M. J. (1957) Fluorometric Method for Measurement of 4-Amino-10-methyl Pteroylglutamic Acid (Amethopterin) in Plasma. *J. Pharmacol.*, **120**, 1.

FREEMAN-NARROD, M., GERSTLEY, B. J., ENGSTROM, P. F. & BORNSTEIN, R. S. (1975) Comparison of Serum Concentrations of Methotrexate after Various Routes of Administration. *Cancer, N.Y.*, **36**, 1619.

FREI, E., JAFFE, N., TATTERSALL, M. H. N., PITMAN, S. & PARKER, L. (1975) New Approaches to Cancer Chemotherapy with Methotrexate. *New Engl. J. Med.*, **292**, 846.

HENDERSON, E. S., ADAMSON, R. H., DENHAM, C. & OLIVERIO, V. T. (1965*a*) The Metabolic Fate of Tritiated Methotrexate. I. Absorption, Excretion and Distribution in Mice, Rats, Dogs and Monkeys. *Cancer Res.*, **25**, 1008.

HENDERSON, E. S., ADAMSON, R. H. & OLIVERIO, V. T. (1965*b*) The Metabolic Fate of Tritiated Methotrexate. II. Absorption and Excretion in Man. *Cancer Res.*, **25**, 1018.

HENDEL, J., SAREK, L. J. & HVIDBERG, E. F. (1976) Rapid Radioimmunoassay for Methotrexate in Biological Fluids. *Clin. Chem.*, **22**, 813.

HUFFMAN, D. F., WAN, S. H., AZARNOFF, D. L. & HOOGSTRATEN, B. (1973) Pharmacokinetics of Methotrexate. *Clin. Pharmac. Therap.*, **14**, 572.

JACOBS, S. A., STOLLER, R. G., CHABNER, B. A. & JOHNS, D. G. (1976) 7-Hydroxymethotrexate as a Urinary Metabolite in Human Subjects and Rhesus Monkeys Receiving High Dose Methotrexate. *J. clin. Invest.*, **57**, 534.

JATON, J. & UNGAR-WARON, H. (1967) Antibodies to Folic Acid and Methotrexate Obtained with Conjugates of Synthetic Polypeptides. *Archs. Biochem. Biophys.*, **122**, 157.

JOHNS, D. G. & VALERINO, D. M. (1971) Metabolism of Folate Antagonists. *Ann. N.Y. Acad. Sci.*, **186**, 378.

KINKADE, J. M., VOGLER, W. R. & DAYTON, P. G. (1974) Plasma Levels of Methotrexate in Cancer Patients as Studied by an Improved Spectrofluorometric Method. *Biochem. Med.*, **10**, 337.

LEVINE, L. & POWERS, E. (1974) Radioimmunoassay for Methotrexate. *Res. Comm. Chem. Path. Pharm.*, **9**, 543.

LOEFFLER, L. J., BLUM, M. R. & NELSON, M. A. (1976) A Radioimmunoassay for Methotrexate and its Comparison with Spectrofluorimetric Procedures. *Cancer Res.*, **36**, 3306.

RASO, V. & SCHREIBER, R. (1975) A Rapid and Specific Radioimmunoassay for Methotrexate. *Cancer Res.*, **35**, 1407.

ROBINSON, J. D., MORRIS, B. A. & MARKS, V. (1975) Development of a Radioimmunoassay for Etorphine. *Res. Comm. Chem. Path. Pharmac.*, **10**, 1.

ROBINSON, J. D., MORRIS, B. A., PIALL, E. M., AHERNE, G. W. & MARKS, V. (1975) The Use of Rats in the Screening of Drug–Protein Conjugates for Immunoreactivity. In: *Radioimmunoassay in Clinical Biochemistry*, Ed. C. A. Pasternak, Oxford: Heyden. p. 91.

ROTHENBERG, S. P. (1965) A Radioenzymatic Assay for Folic Acid. *Nature, Lond.*, **206**, 1154.

SHAPIRO, W. R., YOUNG, D. F. & MEHLA, B. M. (1975) Methotrexate Distribution in Cerebrospinal Fluid after Intravenous, Ventricular and Lumbar Injections. *New Engl. J. Med.*, **293**, 161.

VALERINO, D. M. (1972) Studies on the Metabolism of Methotrexate. II. Isolation and Identification of Several Unconjugated Amino-pteridines as Metabolites in the Rat. *Res. Comm. Chem. Path. Pharmac.*, **4**, 529.

WERKHEISER, W. C. (1961) Specific Binding of 4-Amino Folic Acid Analogues by Folic Acid Reductase. *J. biol. Chem.*, **236**, 888.